# Systems Biology Approaches for Therapeutics Development Against COVID-19

**DOI:** 10.3389/fcimb.2020.560240

**Published:** 2020-10-28

**Authors:** Shweta Jaiswal, Mohit Kumar, Mandeep, Sunita, Yogendra Singh, Pratyoosh Shukla

**Affiliations:** ^1^ Enzyme Technology and Protein Bioinformatics Laboratory, Department of Microbiology, Maharshi Dayanand University, Rohtak, India; ^2^ Soil Microbial Ecology and Environmental Toxicology Laboratory, Department of Zoology, University of Delhi, Delhi, India; ^3^ Department of Zoology, Hindu College, University of Delhi, Delhi, India; ^4^ Bacterial Pathogenesis Laboratory, Department of Zoology, University of Delhi, Delhi, India

**Keywords:** systems biology, multiomics, *in silico*, database (DB), COVID-19, coronavirus, pathogenicity

## Abstract

Understanding the systems biology approaches for promoting the development of new therapeutic drugs is attaining importance nowadays. The threat of COVID-19 outbreak needs to be vanished for global welfare, and every section of research is focusing on it. There is an opportunity for finding new, quick, and accurate tools for developing treatment options, including the vaccine against COVID-19. The review at this moment covers various aspects of pathogenesis and host factors for exploring the virus target and developing suitable therapeutic solutions through systems biology tools. Furthermore, this review also covers the extensive details of multiomics tools *i.e.*, transcriptomics, proteomics, genomics, lipidomics, immunomics, and *in silico* computational modeling aiming towards the study of host–virus interactions in search of therapeutic targets against the COVID-19.

## Introduction

COVID-19 (coronavirus disease 2019) outbreak is caused by an animal virus belonging to the family Coronaviridae (Ahmed et al., 2020; Cascella et al., 2020). This animal virus is transmitted to humans and causes severe respiratory syndromes (Mohd et al., 2016; Fung et al., 2020). The associated syndromes are Middle East respiratory syndrome (MERS) (Wernery et al., 2017), severe acute respiratory syndrome (SARS) (Lai et al., 2020), acute respiratory distress syndrome (ARDS) (Wu et al., 2020b), and most recently coronavirus disease (COVID-19). With the outbreaks related to the above mentioned coronavirus related syndromes, it is evident that human pathogenic coronavirus related mutants and strains occur and emerge from infected animal livestock during the past decade (Deng and Peng, 2020). In the present situation, COVID-19 has been spread globally (Khachfe et al., 2020). This crisis started in China in December 2019. The Wuhan market (China) was associated with animals and their meat products for domestic cooking purposes. The consumption of coronavirus infected meat products by nearby local people is the starting point of the pandemic (Schwartz and Graham, 2020). Within one month approximately, 9,066 positive coronavirus infected cases were found, and 213 patients died till January 2020 (Riou and Althaus, 2020). Moreover, the cases increased continuously at multiple rates around the world, leading to a global health emergency. The coronavirus outbreak has proven a threat to humanity. The coronavirus associated with COVID-19 shows 75–80% similarity with the severe acute respiratory syndrome coronavirus (SARS-CoV) and is more directly connected to numerous bat coronavirus. Unlike other coronaviruses, COVID-19 grows better in epithelial cells of human airway rather than in the cultured cells in the laboratory. It uses human angiotensin-converting enzyme 2 as its cellular receptor, so the infection is transmitted only after the infection of the lower respiratory tract (Perlman, 2020). The novel coronavirus causes severe respiratory disease, COVID-19. The patients suffer from pneumonia. They develop a cold, dry cough and a sudden rise in body temperature (Zhu et al., 2020). Human to human transmission of COVID-19 occurs through respiratory droplets of the sneezed particles or from the close contact of an infected person (Chen, 2020). The WHO (World Health Organization) is currently engaged in managing the pandemic situation with nations around the world by releasing guidelines for health workers. For the novel coronavirus 2019, the unavailability of vaccine tenders the importance of antiviral drugs and therapeutics for pandemic control programs and preventive measures in pandemic reoccurrence (Sohrabi et al., 2020). The current outbreak can be controlled by maintaining social distance and reducing the person to person transmission. The immediate step required to control disease (Archana et al., 2015) outbreak includes isolation, early diagnosis, and other supportive treatment (Czernin et al., 2020). It can also be preventive by maintaining personal hygiene, avoiding crowded places, wearing of fitted masks, and ventilation. Special measures should be taken for the children, old age, and immuno-compromised people as they are more prone to COVID-19 (Marchand-Senécal et al., 2020). The therapeutic drugs available to clinical workers for the treatment of coronavirus infections are only as a temporary option. This new demand gives the opportunity for researchers to save humankind from this menace. The SARS viruses are difficult and quite dangerous to handle *in vivo*, but the information of their genes, proteins, or the RNA acquired by sequencing is simple and easy to handle through artificial intelligence. The expectation from systems biology for therapeutic agent development is mentioned. Furthermore, the role of different components of multiomics is discussed for virulence assessment of coronavirus. Along with the importance of artificial intelligence in generating data for drug development and the requisite of data mining from the database, the *in silico* appeal for host–virus interaction study (*viz*. protein–protein interaction, computational modeling) and vaccine development are also described. Molecular docking studies have been used for the detection of medications to inhibit SARS-CoV-2 spike protein and protease enzyme in the past. Thus, molecular docking can pave the way for computational drug designing, which can further be utilized for the treatment of COVID-19 (Hall and Ji, 2020).

## Pathogenesis and Virulence Strategy

Coronavirus is an enveloped and single-stranded RNA virus. It is classified into four categories: *α*-coronavirus, *β*-coronavirus, *δ*-coronavirus, and *γ*-coronavirus (Yang and Wang, 2020). Earlier, there were six coronaviruses that infect humans and cause diseases. Despite the SARS-CoV and MERS-CoV, COVID-19 is caused by the seventh member of the coronavirus family to infect human, the novel SARS coronavirus and SARS-CoV share almost 79% genome similarity (Dawood, 2020). Like SARS-CoV and MERS-CoV, COVID-19 is considered in the family of *β*-coronavirus (Guo et al., 2020). These two (SARS-CoV and SARS-CoV-2) have identical domains for receptor binding and use angiotensin-converting enzyme 2 (ACE2) as the receptor. Significantly, S protein present on the surface is responsible for the identification of the receptors in the target host, facilitating the entry into the host cell (Hofmann and Pöhlmann, 2004). The binding efficiency of SARS-CoV-2 is ten times higher than the SARS-CoV. This shows that ACE2 could be a possible candidate for treatment (Wang X. et al., 2020). The fact that there is less information about the pathogenesis of SARS-CoV-2 and also that systems biology and omics technology cannot cover every specific cellular or physiological process for hindering the virulence strategy of virus are the main limitations. Patients suffering from COVID-19 have symptoms similar to SARS-CoV and MERS-CoV like fever, fatigue, non-productive coughs, myalgia, pneumonia, and decrease leukocyte count (Daga et al., 2019). Metabolic acidosis showing dysfunction of microcirculation was also observed. Additionally, kidney and liver functions were also affected in some patients. The blood and lower respiratory tract specimen cultures turned out to be negative for bacteria and fungus in 76% sepsis patients in a COVID-19 cohort. Therefore, viral sepsis would be more accurate to describe the clinical manifestations of severe or critically ill COVID-19 patients. Understanding the mechanism of viral sepsis in COVID-19 is warranted for exploring better clinical care for these patients (Zhou et al., 2020).

Therefore the pathogenesis mechanism of SAR-CoV and MERS-CoV will help in understanding the pathogenesis of SARS-CoV-2. Significantly spike proteins are determined to facilitate the entry of the virus into the host body (Xia et al., 2020). They bind to the receptor of the cells like ACE2 and CD209L. Initially, it was reported that the virus enters the cell by the fusion of the virus with the plasma membrane. The proteolytic cleavage of spike protein at position S2′ is essential for membrane fusion following viral infection (Belouzard et al., 2009; Walls et al., 2020). While in MERS-CoV, the membrane fusion was initiated by furin activation. Besides, SARS-CoV-2 uses clathrin-dependent and independent endocytosis methods to enter into the cell (Wang et al., 2008). The virus releases RNA genome into the cell to begin the process of replication. The glycoproteins form to facilitate the formation of the nucleocapsid. The germination of the virus particle takes place in an endoplasmic reticulum golgi intermediate compartment (ERGIC) (Risco et al., 2002). Afterward, the virus particle fused to the plasma membrane to release.

After entering into the host cell, the antigen peptides presented by major histocompatibility complex (MHC) and virus-specific cytotoxic T lymphocytes (CTLs) help in the identification. Therefore, the knowledge of antigen presentation of SARS-CoV-2 will significantly assist in interpreting the pathogenesis of COVID-19 (Prompetchara et al., 2020). As there is less information about antigen presentation for COVID-19, so the information behind SARS-CoV and MERS-CoV will significantly help the researchers in planning the methodologies (Yuen et al., 2020). Mainly SARS-CoV involves MHC I molecules for antigen presentation and also susceptible to different HLA (Human Leukocyte Antigen) polymorphisms. Most of the alleles like HLA-A∗0201, HLA-DR0301, and HLA-Cw1502 help in the protection from SARS disease. The alleles, like HLA-DRB1∗11:01 and HLA-DQB1∗02:01 are more sensitive to MERS-CoV disease (Risco et al., 2002). Nguyen et al., 2020 studied the binding efficiency of HLA and mentioned that HLA-B*15:03 is responsible for maximum binding with conserved peptide of SARS CoV2. Additionally Wang W. et al. (2020) applied next generation sequencing method and found that HLA‐C*07:29 *and* B*15:27 are highly significant in COVID-19 infected patients.

The mannose-binding lectin (MBL) is an important molecule in innate immunity and starts its function before the response of a specific antibody (Ip et al., 2005). People infected with COVID-19 have low level of MBL in their serum as compared to healthy ones. It is observed that MBL is associated with antigen presentation and is also linked with the infection of SARS (Mason and Tarr, 2015). Furthermore, this evidence will be a helping hand in understanding the mechanism of SARS-CoV-2 infection. In comparison with the humoral response, cellular immunity is more significant in the case of coronavirus (Wang F. et al., 2020).

Immune dysfunction such as severe respiratory failure is observed in COVID-19 patients in a case study by Giamarellos-Bourboulis et al. (2020). The macrophage activation syndrome, less human HLA-DR expression along with the reduced number of CD4 lymphocytes, natural killer cells, and CD19 lymphocytes were shown in severe respiratory failure along with sustained production of TNF-α and IL-6 (Magro et al., 2020). The inhibition of HLA-DR expression was performed by plasma of COVID-19 patients, and it could be partially restored *via* IL-6 blocker. Herein, IL-6 based HLA-DR expression is a characteristic feature and deals with hyper inflammation and cytokine production. Generally SARS-CoV-2 causes hyper inflammation by impairing the host immune response and subsequently dearranging the renin–angiotensin–aldosterone system (Henry et al., 2020a). Acute lung injury and coagulopathy were caused by an imbalance in RAAS (renin–angiotensin–aldosterone system) and hyper inflammation. RAAS is an essential hormone system that performs the function of blood pressure regulation and is also helpful in balancing the fluid within the body. Moreover, it would result into fibrinolysis, immunothrombosis, and multiple organ damage (Henry et al., 2020a). The patients in later stage have deteriorated conditions and die within a short period of time because of organ failure and acute respiratory distress syndrome. All these happen due to cytokine storm and it is significant in increasing the symptoms. Cytokine storm was also validated by clinical studies studying critical patients. Hence, the suppression of cytokine storm is another way to treat COVID-19 infected patients (Ye et al., 2020).

### Host Factors

During the 2002–2003 SARS epidemics, the human population got infected from the cross-transmission of civet, raccoon, and Chinese ferret-badger. Initially, the animal handlers got infected from the wet market (Perlman and Netland, 2009). Although they do not have any symptoms of SARS-CoV during detection, their serum gave a high positive result. The infection rate increases when a physician gets infected by treating them, and consequently, the epidemic started. The genetic analysis of the isolated virus reveals its fast rate of adaption in the host cell. It was found in the live animal market and isolated from *Rhinolophus* spp. Hence the virus is transmitted from bats to mammals and then to humans (He et al., 2014). In civets and humans, the virus gets entry through the ACE2 receptor, which was not observed in the case of the bat. Apart from its wild ruminants, canine and feline were also susceptible to contamination with the same virus (Malik et al., 2020).

The study by sequence data analysis and molecular biology reveals that approximately 60 novel bat coronaviruses were found in Africa, North America, Europe, and China (Hu et al., 2015). This strain probably originated from the same source and got diverted based on adaption in a different host. The coronavirus isolated from *Delphinapterus leucas* was also categorized in sub-group, infecting mammals. The spike protein of SARS-CoV and new SARS-CoV-2 shares around 76.5% amino acid identity (Zhang et al., 2020). The coronavirus can quickly enter into the host cells *via* spike protein. The spike protein undergoes cleavage before entering into the target cell. Mostly, SARS-S involves angiotensin-converting enzyme 2 (ACE2) for receptor and also incorporate TMPRSS2, a cellular serine protease that is clinically proved to block the entry of the virus into the cell (Hoffmann et al., 2020). The study about SARS-S/ACE2 elucidated that it contributes an essential role in virus transmission, pathogenesis, and target as a therapeutic agent. SARS-CoV mainly infects pneumocytes and macrophages present in the lungs (Liu J. et al., 2020). Apart from lungs, ACE2 was also expressed in the extra-pulmonary surface. SARS-CoV-2 enters the cell by membrane diffusion and slows down the regulation of ACE2 receptor (Qian et al., 2013; Silhol et al., 2020). TMPRSS2 based entry of SARS-CoV-2 has been observed when cleavage of the S1/S2 site is mediated by furin in the virus-infected cell (Coutard et al., 2020). Thus, it can be deciphered that TMPRSS2 is a vital host factor responsible for COVID-19 spread like coronavirus and influenza A viruses. Iwata-Yoshikawa et al. (2019) reported that TMPRSS2 is a drug target as it contributes to the development of homeostasis. The Camostat mesylate is a serine protease inhibitor that can be sufficient to block the function of TMPRSS2, and Japan approved it for humans (D’Amico et al., 2020). Therefore, the above-discussed compounds possess antiviral activity and were suggested for the treatment of COVID-19. In SARS-CoV, lysosomotropic agents make the infection more severe and sensitive by disturbing endosomal pH (Docea et al., 2020). In SARS-CoV infection, mainly protease treatment nullifies the effect of blocking mediated by lysosomotropic-agent. Cathepsin L, which is an endosomal protease, can also block the SARS-CoV-2 infection (Liu T. et al., 2020) and is also significant in triggering the membrane fusion and is one of the extraordinary phenomena in the pathogenesis of SARS-CoV (Smieszek et al., 2020). The age is also an important factor in COVID-19 infection. In a clinical research, Lighter et al. (2020) reported that people more than sixty years are at higher risk of the infection. However, Jin et al., in a study found that men are more prone to the infection and their surviving potency is lower than females (Jin et al., 2020). Conditions of the patient become more severe if they are suffering from other diseases like hypertension, respiratory disorder, and cardiovascular diseases. Involvement of these diseases may enhance the mortality in this case (Yang Y. et al., 2020).

### Drugs Available and Treatment

**Figure 1:**
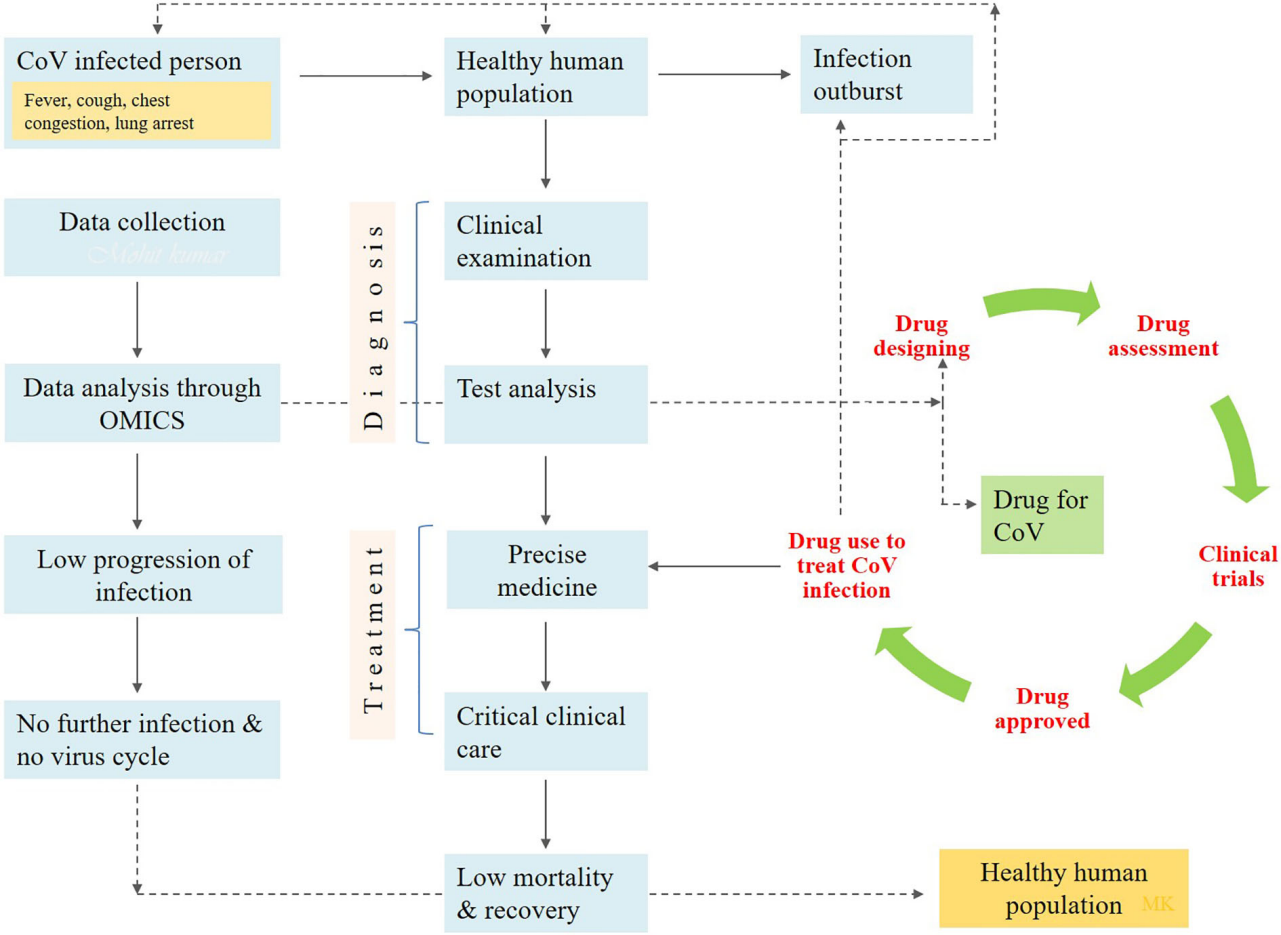
The diagrammatic flow chart showing the implementation of drug to prevent the human health from COVID-19 infection.

Presently, no specific drug and vaccine are available to combat COVID-19 infection. Although, various drug compounds are in the experimental and trial pipelines till now. EIDD-2801 is one of the potential clinical candidates for seasonal and pandemic influenza (Hampton, 2020). Hence, it can be suggested as a potential drug to treat COVID-19 only after clinical trials. Besides the implementation of neuraminidase inhibitors, RNA synthesis inhibitors, Lopinavir/Ritonavir, and peptide (EK1) can be used for its treatment (Rothan and Byrareddy, 2020). However, they are not sufficient to combat SARS-CoV-2 infection. According to recent reports, the antiviral remdesivir and chloroquine have safe records and can be efficiently implemented to treat COVID-19 infection (Zhang and Liu, 2020). Initially, it was suggested by the Washington Department of Health to use remdesivir intravenously to protect against COVID-19. Remdesivir is sufficient to block RNA synthesis by targeting RNA-dependent RNA polymerase and is being potentially used as an antiviral drug for the various RNA viruses (Patankar, 2020). Subsequently, remdesivir and chloroquine were implanted to treat COVID-19 infection. Favipiravir, ribavirin, and galidesivir are the nucleoside analog to be potentially used. Non-structural proteins *i.e.*, chymotrypsin and papain-like protease, are required for virus replication and host immune response inhibition (Chen Y. W. et al., 2020). Inhibitors against them like cinanserin, flavonoids, and PLP inhibitors can be alternatively used for the treatment of the disease. More novel therapeutic agents are urgently required globally to fight against it. Furthermore, a list of non-specific drugs available to cure COVID-19 infection is mentioned in **Table 1**. Alternatively, some antiviral, *i.e.*, nucleoside analogs and HIV-protease inhibitors, can be used to attenuate coronavirus viral infection. The treatment course included various drugs such as oseltamivir, lopinavir, and ritonavir. Along with the intravenous administration of ganciclovir, the patients are advised to take them twice a day for 3 to 14 days (Tobaiqy et al., 2020). However, in first-line treatment, paracetamol is used to treat fever, and expectorants (guaifenesin) should be given for non-productive cough. Oxygen therapy is required in critical conditions like severe acute respiratory infection and hypoxemia. The oxygen supply rate is 5 L/min in most of the children and non-pregnant women. However, in pregnant women, the supply rate is more than 92–95% (Huang et al., 2020). Patients suffering from AKI (acute kidney injury) should be subjected to renal replacement therapy. Antibiotic therapy starts within one hour after the confirmation of the symptoms. The bacterial and fungal infections can occur in the patients in the late and middle stages of diseases. So, it should be advisable to follow conventional and rational antibiotics followed as precision medicine applicable to a patient’s condition under critical care units (**Figure 1**). The implementation of IFN-α and lopinavir/ritonavir is recommended by the National Health Commission of the People’s Republic of China (Wang and Zhu, 2020). The implementation of the above medicines reduces the mortality rate in SARS infected patients (Chu et al., 2004). Methylprednisolone may also consider the children for a maximum of five days (Mouton et al., 2020). The patients suffering from the severe immune response are advised to take glucocorticoids. Different vaccine types such as subunit vaccines, attenuated viruses, and viral vector-based vaccines, inactivated viruses, DNA vaccines, and recombinant proteins can probably be used to cure COVID-19 infection (Saif, 2020). Trials on animal models are conducted to study the biological behavior of COVID-19. Presently, researchers worldwide are working for the development of a non-human primate model to know the mechanism of its interaction with the host (Chen et al., 2019).

**Table 1:** List of proposed therapeutic agents for the treatment of COVID-19.

S.No.	Proposed Drugs	Action of mechanism	References
1.	Ribavirin	Inhibit RNA synthesis	Khalili et al., 2020
2.	Ritonavir	Inhibit HIV viral proteinase enzyme	Cheng et al., 2020
3.	Methylprednisolone	Activation of specific nuclear receptors, alter gene expression and inhibit cytokine production	Yang Y. et al., 2020
4.	Hydrocortisone	Inhibitor of neutrophil apoptosis, phospholipase A2, NF-Kappa B	Russell et al., 2020
5.	Mycophenolate mofetil	Inosine monophosphate dehydrogenase inhibitor	Seminari et al., 2020
6.	Hexamethyleneamiloride	Inhibitor of HCoV-229E and inhibit replication of parent coronaviruses	Farag et al., 2020
7.	Chloroquine	Increase endosomal pH for virus/cell fusion, and interfere with glycosylation of cellular receptors of SARS-CoV	Wang M. et al., 2020
8.	Chlorpromazine	Inhibit clathrin-mediated endocytosis	Zumla et al., 2020
9.	Amodiaquinedihydrochloride	Heme polymerase activity inhibition	Lee et al., 2020
10.	Lycorine	Cell division inhibition, antineoplastic and antiviral	Liu T. et al., 2020
11.	Emetine	RNA, DNA, and protein synthesis inhibition, antiviral	Bleasel and Peterson, 2020
12.	Mycophenolic acid	Inhibitor of inosine-5′-monophosphate dehydrogenase.	Weißbarth et al., 2020
13.	Pyrviniumpamoate	Mitochondrial respiration complex 1 inhibition and suppression of unfolded protein response	Jeon et al., 2020
14.	Remedisivir	Nucleic acid inhibition	Choy et al., 2020

### Development of Novel Biomarkers

The COVID-19 patients are generally diagnosed based on the clinical data. This takes time as the symptoms are generated after the infection sets deeper in the lungs. In such a situation, it becomes difficult for healthcare workers to treat the patients speedily. Biomarker identification provides an advantage over clinical diagnosis. Ulhaq and Soraya identified interleukin-6 (IL-6) as a potential biomarker for COVID-19 infection. As COVID-19 is linked with swift replication and a propensity to infect the lower respiratory tract, so it results in an increased response of IL-6-promoted severe respiratory distress. Thus, the levels of IL-6 can be linked to the disease progression in patients which can prove helpful in further treatment (Ulhaq and Soraya, 2020). C-reactive protein (CRP) is produced by the liver and induced by a range of inflammatory intermediaries such as IL-6. Regardless of its non-specificity, this acute phase reactant is used clinically as a biomarker for different inflammatory situations; an augmentation in CRP intensity is related with an increase in severity of disease. Lactate dehydrogenase is related to severity of pneumonia. Significant rise in LDH levels was observed among refractory COVID-19 patients. The COVID-19 infection leads to thrombocytopenia so platelet count is also a reliable marker for diagnosis of disease severity (Kermali et al., 2020). The Hematologic biomarkers include increase in leukocyte count as a distinguishing factor among infected and non-infected people. In a meta-analysis (Yang J . et al., 2020) carried out by Henry and colleagues in 2020, they found that among 2,984 COVID-19 patients there was significant difference in leukocyte count among severe and non-severe patients (Henry et al., 2020b). According to Bernheim et al., 2020, chest tomography (CT)/X-ray imaging is also a vital component to diagnose COVID-19 suspects when the number is large for diagnosis. In a study they found that CT scan of 56% infected individuals of COVID 19 came normal. Thus it has limited sensitivity and negative predictive value in the early stage of infection (Bernheim et al., 2020).

## The Expectation From Systems Biology for Therapeutic Agents

With the advent of next-generation technologies, the systems biology (Hu et al., 2020; Tang et al., 2020) is applied for the assessment of microbial virulence and associated pathogens (Pezeshki et al., 2019; Çakır et al., 2020; Eckhardt et al., 2020). Information and risk assessment of novel pathogens that emerged with time due to mutations, recombinants, horizontal/vertical gene transfer and reoccurrence of outbreaks need to be presented into the databases (McCormick, 2003; Pybus et al., 2007; Escobedo-Bonilla, 2013; Hartzell and Blaylock, 2014; Kaiser et al., 2015; Deneke et al., 2017; Liu et al., 2019; Olanya et al., 2019).

**Figure 2:**
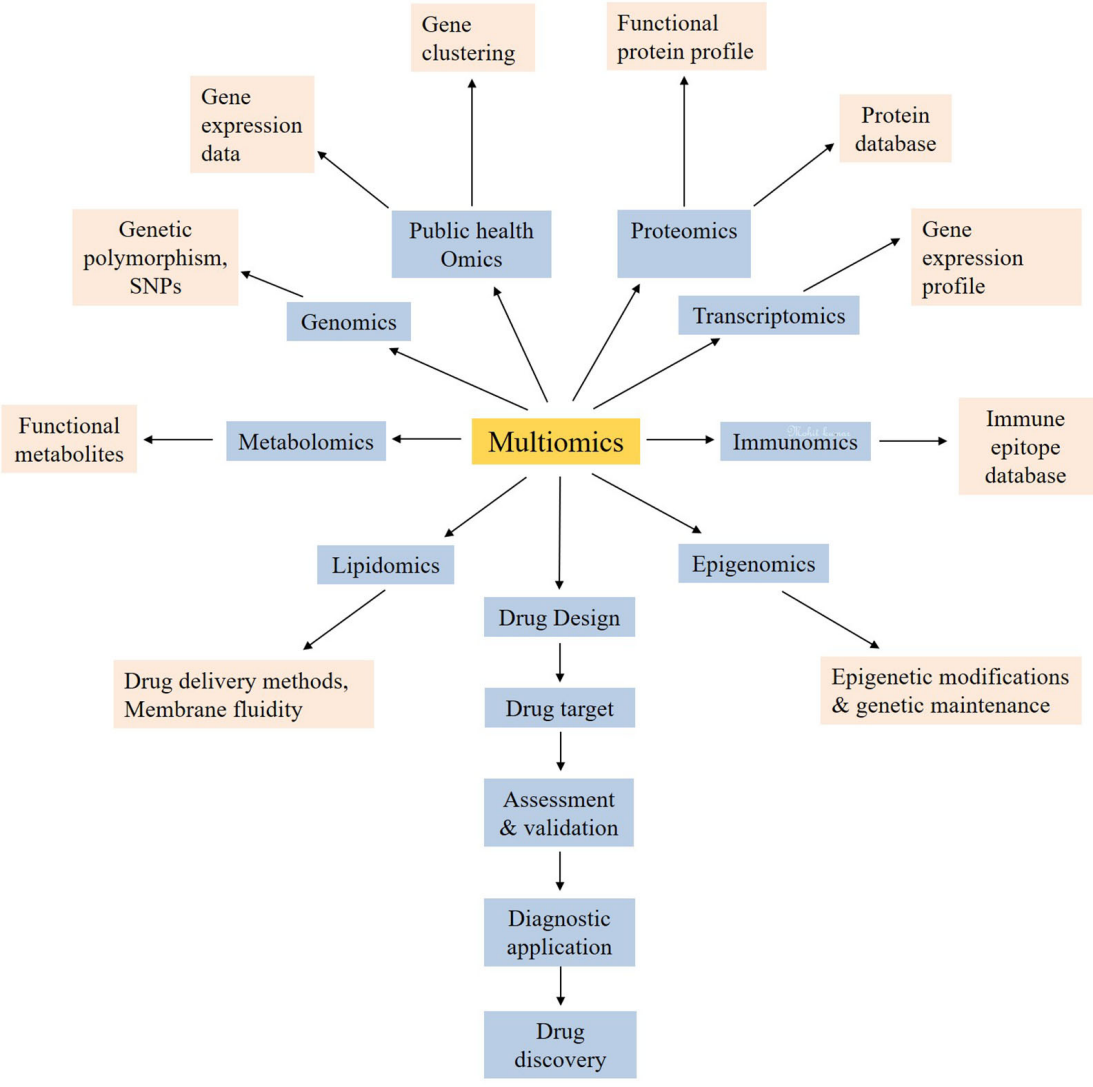
The multiomics approach for the systemic use of technologies for the development of new drug targets for coronavirus.

This update to the existing databases of newly emerged or novel pathogens create a challenge and an opportunity for multiomics experts to collect information/data, reunite and organize new standard datasets/databases or update the already existing databases (Kong et al., 2006; Winnenburg et al., 2007; Schumacher et al., 2014; Xie et al., 2017; Bloch and Bailin, 2019; Dong et al., 2019; Duncan et al., 2019; Yan et al., 2019; Chan et al., 2020). The new information will help the clinical and medical researchers to plan research methodologies for therapeutic and drug development. For instance, the novel coronavirus is a newly emerged pathogen which is creating nuisance to humankind. It is estimated that, globally, approximately 0.2 million patients out of thirteen million infected cases died with coronavirus infection, and the rates are still increasing (Baud et al., 2020; Chaurasiya et al., 2020; Kobayashi et al., 2020). The risk, hazards, and exposure characterization and assessment (Njage et al., 2019; Perera et al., 2019; Pavelić et al., 2020) are essential for building the background knowledge to evaluate the possibilities of novel therapeutics and drugs discovery against coronavirus. The systems biology majorly consists of multiomics, databases, and *in silico* studies (Arora and Singh, 2018). *In silico* approach is mainly dependent on computational designing (Koutsoukas et al., 2011; Lavecchia and Cerchia, 2016) and analyzing the interaction of proteins (Bultinck et al., 2012; Oany et al., 2014). The database mining also contributes to the *in silico* approach (Loging et al., 2007; Rao et al., 2014). As prerequisite to the database (Boissel et al., 2004; Nagata and Pastan, 2009), researching the information is required for the practical planning of methodologies (Wu et al., 2011). The study of heritable phenotypic changes, called as epigenomics, is supposed to play an important role in understanding the mortality rate among black and white individuals (Holmes et al., 2020). These changes may be inherited by the cell system as the memory (Holliday, 1987). The covalent modifications on lysine acetylation, lysine methylation, arginine methylation, serine, and threonine phosphorylation, lysine ubiquitination are the major epigenetic mechanisms which can determine the many genetic and phenotypic modulations (Holliday, 1987; Zhang et al., 2019). The heterogeneity in treatment success is also believed to be influenced by these epigenomic changes (Holmes et al., 2020). As the corona virus targets on the lung cells (Conti et al., 2020), the epigenetic control of ACE2 in the lungs cannot be denied. As Woo and Alenghat highlighted the regulation of transcription during host–microbe interaction under the epigenetic modifications, it may be possible that the rate of transcription of virus may be negatively influenced under genetic environmental pressure *e.g.* strong immune cells. Hence, the more epigenetic exploration is needed to prevent the corona virus infection (Woo and Alenghat, 2017). DNA methylation may be one of the major factors in providing fewer sites for attack of the viral genetic material. The components related to biological entities like DNA, RNA, proteins, metabolites (Jacob et al., 2019; MacMullan et al., 2019) correspond to genomics, proteomics, and metabolomics, respectively (Yan et al., 2019; Jenkins and Orsburn, 2020). Various other systems biology approaches which can be utilized for the development of a potent drug against COVID-19 include immunomics, host lipid omics, public health omics and quantitative dynamic omics. This multiomics approach (**Figure 2**) is also taking attention to therapeutic development (Donovan et al., 2019; Lee and Ruppin, 2019).

### Host Virus Interaction Study by Computational Tools—*In Silico* Approach

**Figure 3:**
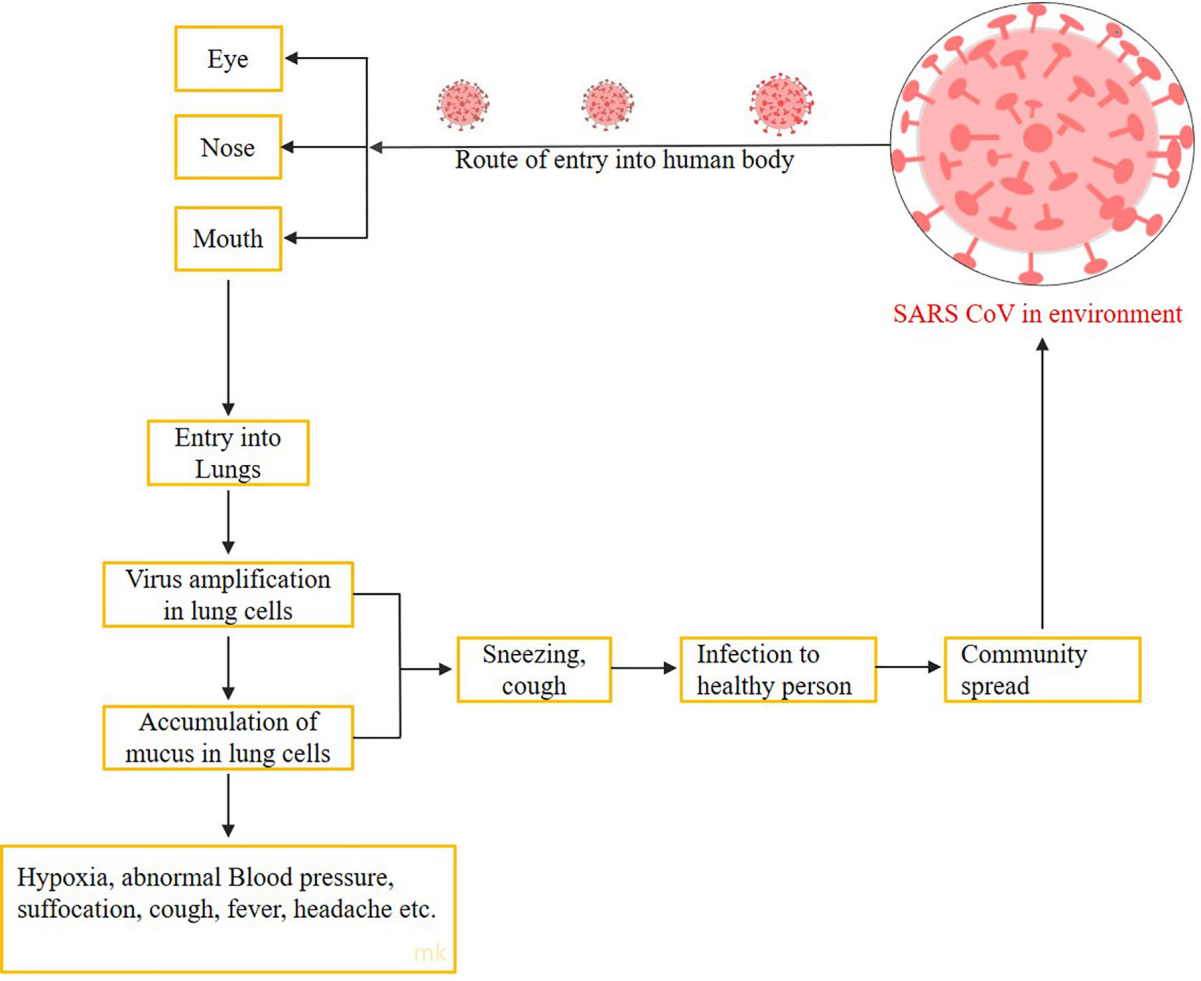
The schematic diagram showing the entry routes and spread of COVID-19.

The interaction of any protein with its receptors always depends on the receptor-binding domains on the proteins (Lan et al., 2020a). These receptor binding domain recognizes their interactive sequences on the receptors and binds them with mostly non-covalent bonds in the human system. However, the SARS-Cov-2 viruses mainly attack the mucus membranes of the human system as their first site of attachment, but they are then bound to their receptors, *i.e.*, angiotensin-converting enzyme 2 (ACE2), and finally helps the viruses to come inside the host cells (Lan et al., 2020b). Moreover, after entering the cell, they start their replication with the help of replication proteins and followed by multiplication steps, as shown in schematic **Figure 3**.

During the multiplication and their amplification steps, SARS-CoV-2 are responsible for the release of some proteases, which leads to the generation of reactive oxygen species (ROS) in the host cell (Chen Y. et al., 2020; Nasi et al., 2020). These ROS are toxic to the cell system and its environment. On the other hand, host defense mechanism activates in the form of their immune system, and the free-flowing neutrophils of the blood reached the target site, the lungs (mainly in the alveoli, where the severe acute respiratory virus reached after attaching the nasal, oral or mucus surfaces). In addition to neutrophils, monocytes differentiate into specific tissue macrophages, in response to the infection. However, neutrophils, basophils, monocyte *etc*. are myeloid progenitor cells and are parts of the innate immunity. These cells, upregulated in any foreign particle, enter into the human body, specifically the virus (van de Laar et al., 2016). The release of cytokines is also supposed to be initiated through adaptive and innate immunity cell signaling. In response to toxicity and foreign particles, the metabolism of the macrophages fluctuates, and they tend to release the inflammatory cytokines which finally affects the molecular signaling of responsive target cells and their neighbor cells. When the normal cellular signaling fluctuates either by ROS stress or by inflammatory cytokines resulting from the SARS-CoV-2 entering the host system, they lead to mucus accumulation in the lungs, abnormal and painful breathing, abnormal BP, *etc.* Here, a small study through computational biology and *in silico* tools for a better understanding of host–virus interaction is designed.

The *Homo sapiens* angiotensin I-converting enzyme 2 (ACE2), transcript variant 1, mRNA of Human origin, with a length of 3,339 bp, was extracted from the NCBI with the accession number NM_001371415.1. The mRNA was converted to the protein sequence by ExPASy; its 5–3 and 3–5 predicted sequences were found. Further, through protein blast, angiotensin-converting enzyme 2 precursor was finalized for the study based on its 100% identity with our query sequence. The accession number of the angiotensin-converting enzyme 2 precursor was NP_001358344.1, and this was the only protein showing 100% identity with our ExPASy query sequence.

**Figure 4:**
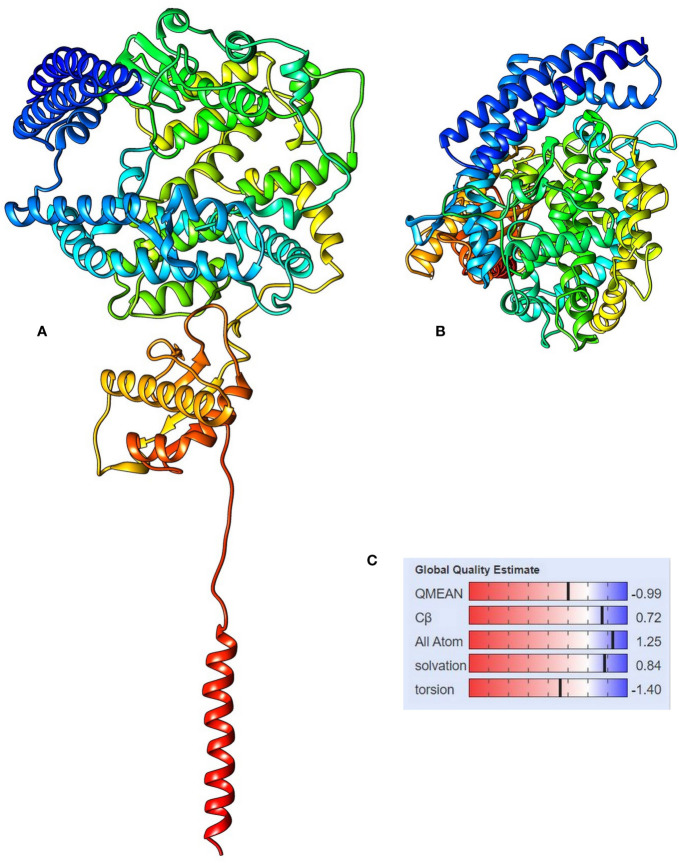
The 3D structure of angiotensin-converting enzyme 2 isoform X1, **(A)** side view and **(B)** top view, and **(C)** the QMEAN value for the protein model showing the statistical representation of the protein model quality.

The 3D modeling of the protein was done using SWISS-MODEL. The obtained protein model was a monomer containing ligands, as N-acetyl-D-glucosamine and Zinc. The template used by the SWISS-MODEL (**Table 2**) for model prediction was 6m17 PDB, and it showed the 100% sequence identity coverage with the predicted model, and that is angiotensin-converting enzyme 2. Now, interestingly, this ACE2 showed to be responsible for acting as a receptor for the coronavirus entry to the cells. The QMEAN value for the predicted protein model was −0.99 and is supposed to be of good quality to be used for the study. **Figure 4** depicts the overall protein 3D model with side and top views, along with its QMEAN value to examine its quality. The Quality of the predicted model was also validated through the ProSA web server, based on NMR and X-ray data. For further validation of the protein model, the ProQ webserver was used, and it predicts the model of extreme quality was good based on the LG score. The Ramachandran plot assessment also suggests its 98% amino acids in the favored region of the plot.

**Table 2:** *In silico* tools used in present study (Protein-protein/Protein-ligand study for drug target and protein target inhibition).

S. No.	*In silico* tool	Function	References
1.	Chemdraw	Ligand structure analysis	Cousins, 2011
2.	SWISS-MODEL	Protein structure homology modeling	Waterhouse et al., 2018
3.	ERRAT	Protein structure validation	Colovos and Yeates, 1993
4.	ProSA	Protein structure validation	Wiederstein and Sippl, 2007
5.	ProQ	Protein structure validation	Wallner, 2005
6.	RAMPAGE	Protein structure validation	Lovell et al., 2003
7.	UCSF Chimera	Protein analysis	Dromey, 1996
8.	PyMOL	Protein analysis	DeLano, 2002
9.	AutoDock	Molecular docking	Norgan et al., 2011
10.	Schrödinger	Molecular docking	Schrodinger, 2011

**Figure 5:**
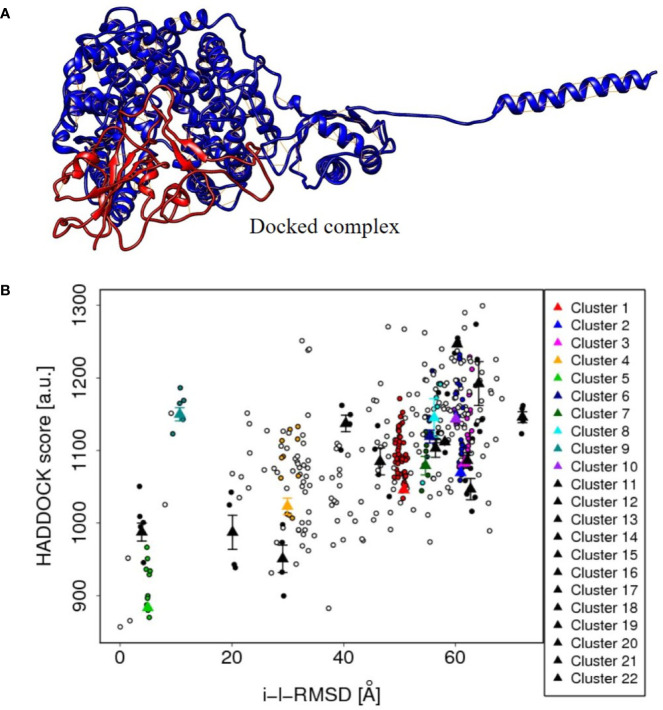
**(A)** The protein-protein docked complex (cluster 5) of ACE2 isoform X1of Human cell (red color), and spike S1 protein (blue color) of corona virus, showing the close interaction. **(B)** The plot between HADDOCK score and RMSD values of the protein–protein docked clusters, showing the best suitability of cluster 5 in the plot and hence to be used to depict the interaction results of ACE2 and spike protein.

Similarly, the spike S1 protein of the coronavirus (source organism; Wuhan seafood market, *Pneumonia virus*) was downloaded from the RCSB protein data bank with the PDB ID, 6M17 (Yan et al., 2020). It was present as a 2019-nCoV RBD/ACE-B0AT1 complex in its PDB format, but to check the protein–protein interaction, the PDB file was modified except for one of the SARS-CoV-2 receptor binding domain. The 3D structure of the protein is shown in **Figure 5**.

The tentative protein–protein interactions are crucial to understand further mechanisms, and a preview of protein–protein docked complex is presented in **Table 3** (van de Laar et al., 2016). Among all the docked complexes, the best structure of cluster 5 complex was depicted in **Figure 5** for its molecular interactions. The cluster 5 has the best HADDOCK score (883.4 +/– 9.7), the lowest Van der Waals energy (–171.7 +/– 9.8), and the Z-score (−2.2). This gives a good understanding towards future tools for understanding such interactions.

**Table 3:** The docking approaches between ACE2 isoform X1 and spike S1 protein of the corona virus by HADDOCK.

S.No. S.No.	Cluster	Haddock score	Vander Waals energy	Z-score
1.	5	883.4 +/− 9.7	−171.7 +/− 9.8	−2.2
2.	22	950.7 +/− 37.6	−141.7 +/− 13.4	−1.1
3.	21	987.2 +/− 46.9	−114.0 +/− 11.9	−0.5
4.	14	987.5 +/− 24.8	−139.6 +/− 11.2	−0.5
5.	4	1,023.0 +/− 22.6	−115.2 +/− 5.7	−0.1

#### Role in Vaccine Development

Vaccine development is in demand due to the increasing rate of mortality and morbidity of COVID-19 infection. Vaccines play an important role in the reduction of toxicity and the elimination of diseases (Dubey et al., 2018; Amanat and Krammer, 2020). The conventional technologies of vaccine designed have many limitations, including time consumption, laborious, costly, and many more. Perhaps somehow, the involvement of *in silico* tools is sufficient to overcome the mentioned limitations (Rauch et al., 2018). Immunoinformatics approach like reverse vaccinology, epitope prediction, rational vaccinology, and structural vaccinology is advantageous to be used for the designing of vaccines (Kazi et al., 2018). Moreover, side chain and backbone modeling are significant in designing the antibody structure to act as vaccine target. SCWRL and SCAP are the *in silico* tools used to identify the mutation in the proteins (He et al., 2015). RAMBLE and RAPER are the additional software applied for side chain prediction analysis. Multivant scaffolding is another tool applied for the designing of potential epitope (He et al., 2015). However, an epitope can be grafted by using multigraft interface technology. ORF-FINDER, GS FINDER, and GLIMMER are some of the *in silico* tools that performed the screening of ORFs and selected the most immunogenic peptide that alternatively help in vaccine designing (Davies and Darren, 2007).

Structural biology is an important area of immunoinformatics and the basis for the structure of proteins. On the basis of structure, rational vaccinology analyzes the structure of novel protein antigens that will be targeted as potential vaccine candidates (Hegde et al., 2018). Rational technology of vaccine design was significantly used against viral pathogens like influenza, HIV (Van Regenmortel, 2019) and Hepatitis C (He and Zhu, 2015). Furthermore, approaching systems biology enhances the understanding of host–pathogen interaction and also develops adjuvant that provides long-lasting immunity. Vaxijen is another important computation tool that contributes to vaccine development. It is an online software that uses the alignment-free approach to predict the antigenic nature of the proteins (Sinha and Shukla, 2019; Bappy et al., 2020). Majorly the critical process in vaccine development is the identification of epitopes that can be targeted as a vaccine candidate. A number of epitope prediction tools are available that can be significantly used for epitope prediction (Naz et al., 2020). The B-cell and T-cell epitopes of novel SARS-CoV-2 were analyzed by Ahmed et al. by approaching IEDB and other computational tools (Grifoni et al., 2020). Ong et al. (2020) reported the reverse vaccinology and machine learning approach were used to identify the potential vaccine candidate against COVID-19 infection (Ong et al., 2020). As reported by Chen and Wu (2020), ABCpred and BepiPred and IEDB are the epitope prediction tools used for the identification of epitopes in the novel SARS-CoV-2. Besides, the multigraft, multivalent scaffolding, codon optimization, and antibodyomics tools are also helpful in the recognition and construction of potential vaccine candidates (Sunita et al., 2020). However, at present computational tools are the first to be used for vaccine designing of emerging diseases. Later on, these will be validated by experimental studies. Collectively, the actual implementation of these disciplines accelerates the process of vaccine development (Chin et al., 2019).

The Chemdraw software is used to draw the molecular structure of a molecule or compound in the computer and is easy to handle in an offline mode. This gives an immediate and clear sharp image of the structure and can be saved in many file types, *eg*; mol file. The file needs not to be redrawn if any correction has to be added. Hence this provides an immediate output of the structures to be used in any docking study. The software, SWISS-MODEL is designed for the homology modeling of the proteins. The software works easily in windows system and gives the output in a simple manner so as the beginners in the field of systems biology can easily benefit. The protein model quality is also validated on the basis of Q mean score, which is the collective information of many parameters *e.g.* X-ray and NMR. After protein homology modeling, there is a need of the formed structure validation, and this task can be done by many online software applicatioms *e.g.*; ERRAT, ProSA, ProQ, RAMPAGE *etc*. Further, the ProQ also gives its own score called as LG score, for the highest ranked models in protein validation. This model validation can be analyzed based on their scores (software gives different scores more or less similar based on their inbuilt programming).

The Ramachandran plot provides the quantitative data for favored and unfavored regions, which basically used the protein backbone dihedral angles and is necessary for protein validation. The Schrödinger and AutoDock software is used for the molecular docking of protein–ligand or protein–protein. However, both the software programs can be used for the docking, but an expert should be needed to deal with these software programs, and it needs some prior knowledge to work with them. The software programs, UCSF Chimera and PyMOL are used for the molecular structure analysis, and it need some knowledge to handle the software programs correctly. But with basic knowledge of molecules and bioinformatics, the online tools can be handled easily and can give the best results in the drug discovery and exploration of proteins. The HADDOCK online server provides the quantitative description for the protein–protein molecular interaction. It provides many clusters of protein–protein interactions based on the possibility of the bond formations and the analysis involved many parameters *eg*; Haddock score, Z-score. These parameters are necessary for choosing the best cluster for further analysis of the molecular interactions using systems biology approach. Van der Waals interaction is the distance dependent interaction between atoms, and Haddock provides us the series of interactions (strongest to weaker). However, so software can easily give results based on their input algorithm, we have to analyze the parameters for the perfect study.

### Role of Omics Techniques for Virulence Assessment

The MRA (Microbial Risk Assessment) (Brul et al., 2012; Haddad et al., 2018) is a clinical evaluation of virulence associated with pathogen, mainly foodborne pathogens *i.e.*, animal meat and their products (Wassenaar et al., 2007; Franz et al., 2016). Nowadays, the utilization of multiomics datasets for improving and redesigning the role model of microbial risk assessment is being practiced by researchers (Buchanan et al., 2000; denBesten et al., 2018). The dose–response models are designed by clinical researchers for studies, especially for diseases associated with RNA viruses (Gale et al., 2014). This probabilistic approach firstly consists of the evaluation of viral infections *via* oral path, *i.e.*, the host and virus interaction *via* cellular receptors with and without conquering the host defense system and replication in the host cell (Voysey and Brown, 2000; Huang and Haas, 2009), followed by designing the models to study disease progress immediately after infection. The virulence markers are the viral gene sequences representing the viral disease-related traits (Haddad et al., 2018; Liang J. et al., 2020). Therefore, it is observed that the above-discussed appeal must be undertaken for coronavirus probabilistic virulence assessment (Benvenuto et al., 2020). Since dose–response models are particular for RNA viruses thus, the possibility and chances of multiomics application (Zhang et al., 2018) to search the antiviral targets rise for the development of therapeutics and drugs against coronavirus (Fritsch et al., 2018; Jeon et al., 2020).

#### Genomics

Genomics offers the task to reveal the characteristics of drugable genomes (Hopkins and Groom, 2002), which consist of sequences and alignments signifying the virulence trait (Losada et al., 2016; Lee et al., 2017). Data mining from databases can make it possible for the researcher to seek out queries related to coronavirus (Hatcher et al., 2017). For the purposive research regarding the newly emerged virus, the National Institute of Health (NIH) of United States created a resource (http://www.niaid.nih.gov), an integrated surveillance data for supporting researchers in collaboration with various research institutes working on systems biology (Squires et al., 2012; Uyeki et al., 2016). The main aim is to make the comparative genomic research for correlative analysis of coronaviridae family-related genus and strains for the predicted data and annotated genome sequences (Zhu et al., 2016; Liang J. et al., 2020; Randhawa et al., 2020). In this process, the MSA (Multiple Sequence Alignment) signifies the closely related RefSeq constructing the virus ortholog groups with associated protein playing role in virulence (Fumagalli et al., 2010; Chapman et al., 2011; Claytor et al., 2017). Till now, there is very little scientific data and literature available related to coronavirus. The genomics approach is leading and generating the manually curated research data (Brister et al., 2015; Manzoni et al., 2018) about the clinical coronavirus strains aiding the scientific literature country-wise in the above-discussed manner (Huang et al., 2007).

#### Transcriptomics and Metabolomics

Transcriptomics is mainly concerned with gene expression profile (Irigoyen et al., 2016) *i.e.*, by ribosome profiling (Irigoyen et al., 2016), RNA sequencing (Depledge et al., 2019), and high throughput DNA microarrays (Wang et al., 2009). The dose–response models are developed to study the factors affecting gene expression (Hashem et al., 2019) at different concentrations of virulence proteins (Haas, 2020). They also check the mRNA abundance at various levels of infection progression (Albariño et al., 2018). The metabolic enzymes run cellular metabolism (Ahmed Idris et al., 2020). The cellular metabolism is revealed through the intense study of genomics, transcriptomics, and proteomics (Fanos et al., 2020), which are directly and indirectly linked to pathways involved in metabolomics (Haas et al., 2016). The primary significance of metabolomics comes for diagnostic assessment (To et al., 2016). The concentrations of metabolites (Sinha et al., 2019) are detected by NMR (Nuclear Magnetic Resonance), HPLC/MS (High-performance Liquid Chromatography/Mass Spectrometry) (Peng and Liu, 2017). Metabolic profile analysis would reveal the binding and inactivation of metabolites by drugs (Eisfeld et al., 2017) that would arrest further disease progression (Zhao and Lin, 2014). In this manner, transcriptomics and metabolomics increase the possibility for developing therapeutics and drugs against the coronavirus.

#### Proteomics

Proteomics study reveals the functional role of proteins associated with host and pathogen (Zheng et al., 2018). With available resources of database and drug targets obtained by previous studies on influenza virus (Sadewasser et al., 2017), hepatitis C virus (Lupberger et al., 2019), poxvirus (Grossegesse et al., 2017), Nipah virus (Vera-Velasco et al., 2018), *etc.*, it is feasible to develop novel drugs. The studies on GPCR (G-protein coupled receptors) (Sriram and Insel, 2018), ion channels (Lin, 2019; Duncan et al., 2020), and enzymes (Ding et al., 2017) provide the platform for researchers to study drug–target interactions. Zheng and Perlman, in 2018 discussed the importance of proteomics in the host immune system and respiratory virus interaction response. They insighted the landscape proteomics analysis formed by prediction of clinical data and the role of immune response gathered *via* host lipid omics, immunomics, phosphoproteomics, and public health omics (Zheng and Perlman, 2018). The recent study done by Kang et al., 2020, the western blotting, protein categorization, gel digestion, SDS-PAGE analysis, SILAC labeling for protein analysis, protein identification, separation and quantification methodologies for identification of structural proteins of coronavirus, mainly bronchitis virus particles were done. The above-discussed method is likely to be the strategy for finding the novel antiviral against coronavirus.

#### Immunomics

Immunomics is based on the efficiency of the host to eliminate pathogens that enter the human body. The immune system of organisms contains many cellular, molecular, and physical components that provide defense against invasive microorganisms. Dysregulation of immune cells such as inflammatory monocyte–macrophage and type I interferon (IFN) led to the occurrence of lethal pneumonia in mice infected with SARS-CoV (Channappanavar et al., 2016). This indicates that immune cells play a vital role in combating pathogens. The immune memory cells are able to protect the host from the early invasion of respiratory pathogens. Bioinformatics tools and sequence homology can be used to find potential immune targets and designing of a vaccine against COVID-19. Grifoni et al. used the Immune Epitope Database (IEDB) and Analysis Resource for prediction of COVID-19. They used SARS-CoV to predict epitope responses as it shows higher similarity to SARS-CoV-2. They found conserved regions in SARS-CoV and SARS-CoV-2 caused COVID-19. Vaccination approach proposed to target the immune response toward these conserved epitope regions could generate immunity. This will not only protect from *Betacoronaviruses* but also against moderately challenging virus that will emerge in future (Grifoni et al., 2020). Carbohydrates present on the host and viral proteins are potential targets for modulating the immune response. The use of computational tools and integrated omics approaches can lead to the development of vaccines and drugs for such targets of viral infection and receptors (Zheng and Perlman, 2018).

#### Host Lipid Omics

Lipids play a vital role in the interaction of the virus with the host cells. Lipids can act straightly as the receptors or co-factors of entry for viruses at the surface of cell or endosomes. Viral replication complex highly depends on them, and lipids also provide energy for replication of the virus. Lipids can help to order the suitable cellular allocation of viral protein and also the trafficking, assemblage, and liberation of viral particles. Thus, host lipid studies can play indispensable role in understanding virus propagation (Diamond et al., 2010; Das, 2020). Coronaviruses seize intracellular membranes of the host cells to produce fresh partitions called double-membrane vesicles (DMVs). These partitions help in the viral genome amplification. A current study showed that a primary lipid processing enzyme, cytosolic phospholipase A2*α* enzyme (cPLA2*α*), was directly linked with DMVs’ development and replication of coronaviruses (Müller et al., 2018). Coronaviruses require a specific composition of lipids for their replication. If this lipid homeostasis is broken, then the viral replication is affected. Yan and his co-workers found in a study that glycerophospholipids and fatty acids (FAs) were considerably increased in the HCoV-229E-infected cells, and the linoleic acid (LA) to arachidonic acid (AA) metabolism was strikingly disturbed upon infection of HCoV-229E. Exogenous supplementation of LA and AA decreased the replication of coronavirus. They came to the conclusion that there was an upregulation of lipids that were responsible for replication and membrane synthesis. The virus maintains homeostasis for its better replication, but when this homeostasis is broken by supplying lipids from outside the cell, the replication of the virus is disturbed. Thus, lipidomics can provide better treatment strategies if integrated with immunological data (Yan et al., 2019).

#### Public Health Omics

Public health omics takes into consideration the entire kinetic response of the host. In public health omics, the expressions of genes and transcriptome are studied. The interaction and regulation of different transcriptome datasets are studied. It takes into consideration the upregulation and down-regulation of different genes during infection. It takes account of molecular as well as clinical conditions of the host and pathogen. The pathway interaction and response of host are analyzed after the infection by utilizing this methodology. This renders the whole set of the idea in host–pathogen interaction with respect to the time of infection. Such systems biology methods draw attention to the significance of time-related factors in the study of multifactorial diseases such as influenza and coronavirus. A study by Dimitrakopoulou and his group revealed the temporal effect of the influenza virus by studying the interactome and signaling pathways. Their findings cooperatively update the budding area of public health omics and potential clinical trials intended to interpret dynamic host reactions to pathogens (Dimitrakopoulou et al., 2014). This is perhaps the unnoticed field of omics technology, but its application can give better results for understanding the spread of COVID-19. This technique will help in the development of time dependent drug development in case of infection (Pawelek et al., 2012).

## Artificial Intelligence for Data Generation and Drug Development

Artificial intelligence plays a vital role in this global scenario to fight against COVID-19. Artificial intelligence and machine learning techniques have helped to group data of genomic taxonomic classification, detection assay based on CRISPR (Dangi et al., 2018; Vashistha et al., 2018), endurance calculation of patients, and identifying probable drug candidates for COVID-19. Metsky et al. screened SARS-CoV-2 by machine learning designs employing a CRISPR-based virus detection system with high speed and sensitivity (Metsky et al., 2020). Similarly, artificial intelligence can be used for the management of critical patients of COVID-19. Rahmatizadeh et al. applied a three-stage model based on input, process, and output. They took into consideration paraclinical, clinical, epidemiologic data, personalized medicine, diagnosis, risk stratification, treatment, prognosis, and management.

The AI (Artificial Intelligence) approach is helpful in stratifying patients and their timely cure (Rahmatizadeh et al., 2020). Thus, computational tools not only help in virus detection but also in drug development. Wu and his co-workers analyzed the proteins coded by the SARS-CoV-2 virus and modeled them for target prediction. They predicted potential targets and probable drugs against them. They screened 3-chymotrypsin-like protease (3CLpro), spike, RNA-dependent RNA polymerase (RdRp), and papain like protease (PLpro) thoroughly. 78 generally used antiviral drugs and compounds from ZINC database were used for positioning and structural analysis. Thus, *in silico* studies provide drug repositioning to treat COVID-19 (Wu et al., 2020a). A deep-learning based analysis structure of thoracic CT images was built for computerized recognition and observation of COVID-19 patients over time. Swift development of computerized diagnostic systems based on artificial intelligence and machine learning cannot only give improved diagnostic accurately and rapidly, but will also defend healthcare workers by diminishing their contacts with COVID-19 patients (Alimadadi et al., 2020).

## Conclusion and Future Perspective

The present review gives an insight into the applicability of systems biology tools for developing drugs against COVID-19 infection. The ultimate aim is to find the possible viral targets by exploring the pathogenicity and virulence strategy of coronavirus. The promising *in silico* application of molecular interaction and simulation study is done purposely for the understanding of host (human) and virus interaction to plan the future strategies for managing the situations of virus pandemics. It is worthy to mention that omics data and systems biology algorithms can combine data from cytokines, blood cell populations, proteomics, transcriptomics, clinical parameters, and epidemiological data to develop personalized medicine strategies and patient stratification based on omics. Although it is difficult to make the strategies or policies by the non-medical expertise of the administration, with the help of systems biology, the possibilities increase. The molecular docking and simulations study are presented to make it simple and approachable to non-target audience also, to ensure the seriousness of the COVID-19 as a global pandemic. In conclusion, it can be stated that the systems biology can lead based on the obtained sequencing data, to ensure the understanding of molecular and physiological mechanisms/phenomena and definitely help in breaking the coronavirus like epidemic outbreaks in future by potential antiviral drugs acting on target for preventing the associated disease progression and increasing the infected patient’s treatment effectiveness.

## Author Contributions

SJ wrote the first draft of the manuscript with contributions from MK, Mandeep, and Sunita. The final draft was read and edited by YS and PS. All authors contributed to the article and approved the submitted version.

## Funding

PS acknowledges the infrastructural support from the Department of Science and Technology, New Delhi, Govt. of India, through FIST grant (Grant No. 1196/SR/FST/LS-I/2017/4). SJ and Sunita acknowledges Maharshi Dayanand University, Rohtak, India, for providing University Research Scholarship (URS). Mandeep acknowledges the Junior Research Fellowship from CSIR, India (Award No. 09/382(0211)/2019-EMR-I). MK acknowledges the support from Prof. D.K. Singh, DU for his permission to be a part of this manuscript and the Hindu College, DU for Innovation project (IP-2019-20/SC/05 and 06).

## Conflict of Interest

The authors declare that the research was conducted in the absence of any commercial or financial relationships that could be construed as a potential conflict of interest.
